# The Beauty of Sound Waves

**DOI:** 10.24908/pocus.v7i1.15314

**Published:** 2022-04-21

**Authors:** Ai Phi Thuy Ho

**Affiliations:** 1 Cardiology Department, Hospital of Kalnes Norway; 2 CEO and Founder, Norvue

**Keywords:** point of care ultrasound, ultrasound

**Figure 1:**
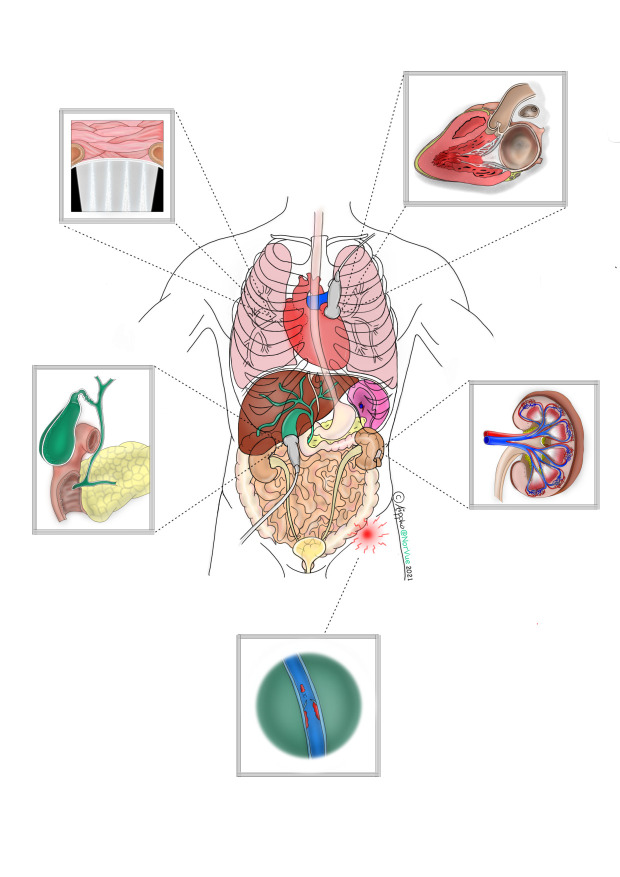
The Beauty of Soundwaves

“The Beauty of Sound Waves” is an artwork representing the anatomy of various organs in the human body that can be detected by ultrasound, either as normal findings or pathology. Made by Ai Phi Thuy Ho. 

## Disclosures: 

None.

